# Extremely Low-Frequency Electromagnetic Fields Increase the Expression of Anagen-Related Molecules in Human Dermal Papilla Cells via GSK-3β/ERK/Akt Signaling Pathway

**DOI:** 10.3390/ijms21030784

**Published:** 2020-01-25

**Authors:** Ga-Eun Ki, Yu-Mi Kim, Han-Moi Lim, Eun-Cheol Lee, Yun-Kyong Choi, Young-Kwon Seo

**Affiliations:** Department of Medical Biotechnology (BK21 Plus team), Dongguk University, Goyang-si 10326, Gyeonggi-do, Korea; gigaeun@naver.com (G.-E.K.); kjmtik@nate.com (Y.-M.K.); gksahl321@gmail.com (H.-M.L.); eunchul90@gmail.com (E.-C.L.); happyyunki@naver.com (Y.-K.C.)

**Keywords:** alopecia, hair follicle dermal papilla cell, electromagnetic field

## Abstract

Despite advances in medical treatments, the proportion of the population suffering from alopecia is increasing, thereby creating a need for new treatments to control hair loss and prevent balding. Human hair follicle dermal papilla cells (hDPCs), a type of specialized fibroblast in the hair bulb, play an essential role in controlling hair growth and in conditions like androgenic alopecia. This study aimed to evaluate the intensity-dependent effect of extremely low-frequency electromagnetic fields (ELF-EMFs) on the expression of anagen-related molecules in hDPCs in vitro. We examined the effect of ELF-EMF on hDPCs to determine whether activation of the GSK-3β/ERK/Akt signaling pathway improved hDPC activation and proliferation; hDPCs were exposed to ELF-EMFs at a frequency of 70 Hz and at intensities ranging from 5 to 100 G, over four days. Various PEMF intensities significantly increased the expression of anagen-related molecules, including collagen IV, laminin, ALP, and versican. In particular, an intensity of 10 G is most potent for promoting the proliferation of hDPC and expression of anagen-related molecules. Moreover, 10 G ELF-EMF significantly increased β-catenin and Wnt3α expression and GSK-3β/ERK/Akt phosphorylation. Our results confirmed that ELF-EMFs enhance hDPC activation and proliferation via the GSK-3β/ERK/Akt signaling pathway, suggesting a potential treatment strategy for alopecia.

## 1. Introduction

Hair disorders such as hair loss (alopecia), androgenic alopecia, or excessive hair growth (hirsutism, hypertrichosis) may impact the social and psychological well-being of an individual [1,2]. Various agents, including herbal and natural extracts [3,4,5], growth factors, cytokines [6,7], placental extract, stem cells [8], conditioned medium, peptides, hormones, lipid nanocarrier, light, physical stimulation [9], androgens and their analogs, stress-serum, and chemotherapeutic agents, have been examined for their hair-growth-modulating effects in hDPCs. Nevertheless, alopecia is an exceedingly prevalent condition which affects men and women of all ages. Only two drugs, namely finasteride and minoxidil, have been approved for hair loss treatment by the US Food and Drug Administration (FDA). However, the effects of the drugs are limited and transient, with unpredictable efficacy and unwanted side effects [10,11,12]. Thus, newer and better therapeutic measures to prevent hair loss and enhance hair growth are urgently needed.

We focused particularly on EMF in this study. EMF has been approved as a noninvasive treatment by the US Food and Drug Administration (FDA). EMF has been verified for stability and is already clinically used [13]. A large number of investigations on extremely low-frequency electromagnetic field (ELF-EMF) exposure, which affects numerous biological functions, such as gene expression [14], cell differentiation [15,16,17], and proliferation [18,19], have been conducted in various in vitro and in vivo models. ELF-EMF is also known to be nontoxic and to prevent inflammatory reactions in epithelial cells and in dermal papilla cells [20].

The hDPCs are promising cell sources for hair regeneration therapy for alopecia patients [7]. Human hair follicles are composed of several different epithelial and dermal papilla cells (hDPCs); hDPCs are a type of specialized fibroblast that is derived from mesenchyme and are located at the base of hair follicles. They play an important role in hair growth and conditions including androgenic alopecia [4,21]. Here, we focused on the biological effects of ELF-EMF on hDPCs. Numerous molecular signals orchestrate hair growth, and the Wnt3α/β-catenin signal is one of the most important of these molecular signals. Wnt3α/β-catenin signaling promotes the development of new hair follicles and is required for the initiation of hair morphogenesis [22,23]. β-catenin in hDPCs is markedly activated during the anagen phase and is important for the maintenance of the anagen phase and hair-cycle regeneration [24]. β-catenin activation in hDPCs can prolong the anagen phase and promote hair growth [25].

β-catenin protein levels are regulated by the Wnt signal. In the absence of Wnt3α, β-catenin is ordinarily phosphorylated in the cytoplasm by GSK3β, a serine/threonine protein kinase encoded by GSK3β. When Wnt protein binds to the cell surface receptors of the Frizzled family, β-catenin destruction is prevented via GSK-3β inhibition. Consequently, β-catenin accumulates in the cytoplasm and moves into the nucleus, where it activates gene expression programs. Thus, GSK-3β is a key protein involved in the regulation of Wnt3α/β-catenin signaling, and GSK-3 inhibition may enhance hair growth by inducing an increase in β-catenin levels in DPCs [23,24,25,26]. Moreover, it was recently shown that Wnt3α/β-catenin signaling is involved in regulation of the expression of extracellular signal-regulated kinase (ERK) and phosphatidylinositol-3-kinase (PI3K)/AKT, as well as in the proliferation of hDPCs [27,28].

In this study, we hypothesized that ELF-EMFs would affect the activity of hair cells, so we examined the effect of ELF-EMFs on hDPCs. In particular, we investigated a frequency effect of EMF for inducing hDPCs growth and activating hair growth-related gene and protein expression. First, hDPCs were isolated from patients and exposed to 70 Hz EMF at intensities of 5, 10, 20, 50, and 100 G and compared with control hDPCs, which had not been exposed to EMF. Moreover, to elucidate the mechanisms, we focused on the β-catenin signaling pathway. Our results demonstrated that β-catenin mediated hDPC activation via GSK-3β inhibition by phosphorylation (Ser9). Our findings revealed that ELF-EMF promoted hair growth capacity and could be potentially used as an alternative therapeutic option in hair loss treatment.

## 2. Results

### 2.1. ELF-EMF Stimulated the Proliferation of hDPCs

**Figure 1:**
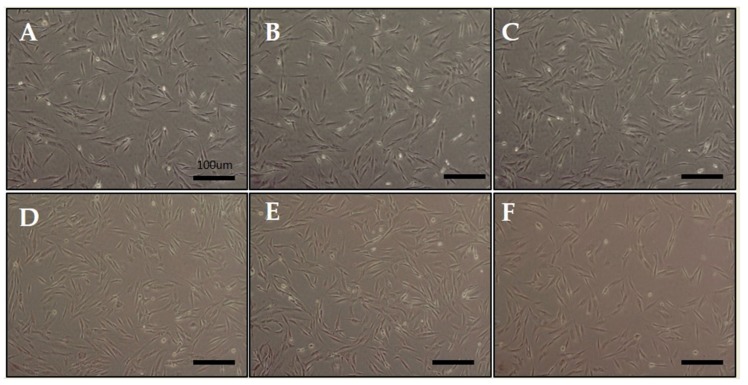
Morphology of human hair follicle dermal papilla cells (hDPCs) after being stimulated by an extremely low-frequency electromagnetic field (ELF-EMF). (**A**) control; (**B**) 5 Gauss (G); (**C**) 10 G; (**D**) 20 G; (**E**) 50 G; and (**F**) 100 G. Original magnification was ×100; bar = 100 μm.

**Figure 2:**
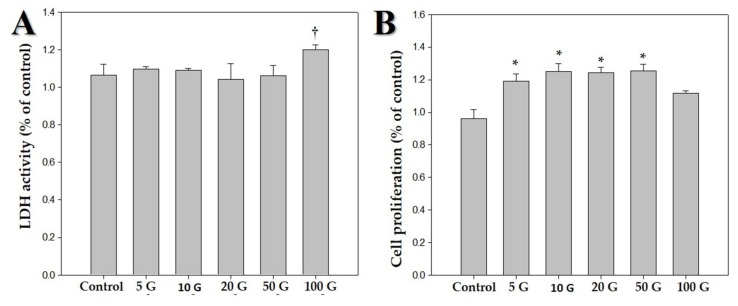
Effects of various intensities of extremely low frequency electromagnetic fields (ELF-EMFs; 5, 10, 20, 50, and 100 G) on human dermal papilla cells (hDPCs). The hDPCs (2 × 10^4^ cells/well) were cultured in Dulbecco’s minimal essential medium for 48 h. (**A**) Cytotoxic effects of ELF-EMFs on hDPCs. Their cytotoxicity was measured by using an LDH assay kit. (**B**) MTT assay was performed for determining viability of hDPCs after treatment with ELF-EMFs († *p* > 0.05, * *p* < 0.05, compared with the control).

We exposed hDPCs to ELF-EMF radiations of various intensities, namely 5, 10, 20, 50, and 100 G, at a frequency of 70 Hz, and performed LDH and MTT assays. Figure 1 shows the morphology of hDPCs after exposure to ELF-EMFs for four days. The morphological features were maintained without cell death. To assess cell damage, LDH assay was performed (Figure 2A). LDH is an oxidative enzyme present in the cell membrane and cytoplasm that is released from cells into culture media after cell damage. The results of this assay revealed that there were no significant differences between the groups of cells exposed to radiation at intensities of 5, 10, 20, and 50 G and the controls. However, there was a small difference between cells exposed to a radiation intensity of 100 G and the controls, but without any statistical significance. Hence, ELF-EMFs appeared to be non-cytotoxic, and thereby did not induce cell damage (Figure 2A). We performed MTT assay for assessing cell proliferation. As opposed to control cells, proliferation of hDPCs significantly increased to ~1.2 fold at intensities of 5, 10, 20, and 50 G (Figure 2B).

### 2.2. ELF-EMF Activates Anagen-Related Molecules in DPCs

**Figure 3:**
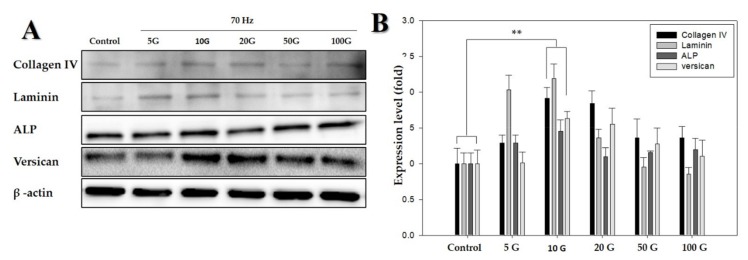
Western blot analysis of proteins detected in human dermal papilla cells (hDPCs) treated with an extremely low-frequency electromagnetic field (ELF-EMF). (**A**) Protein expression of collagen IV, laminin, ALP, and versican, using β-actin as an internal control; (**B**) protein expression level of collagen IV, laminin, ALP, and versican (with the control as the standard). β-actin was used in each lane as an internal control. Each bar represents mean ± standard error of independent experiments performed in triplicate (*n* = 3). Significant differences were determined by the Student’s *t*-test; ** *p* < 0.01.

Following studies on cell morphology, LDH activity assay, and MTT assay, the protein levels of anagen-related proteins (i.e., laminin, ALP, and versican) after exposure to ELF-EMFs over four days were quantified via Western blotting. There were significant differences between cells exposed to ELF-EMFs of different intensities (Figure 3). This revealed that the expression of anagen-related proteins increased more than 1.5-fold in cells exposed to ELF-EMFs compared with the expression in control cells. In particular, an ELF-EMF of 10 G intensity was the most potent in promoting the expression of anagen-related molecules in hDPCs.

### 2.3. ELF-EMF Promotes Wnt3α/β-Catenin Signaling in hDPCs

**Figure 4:**
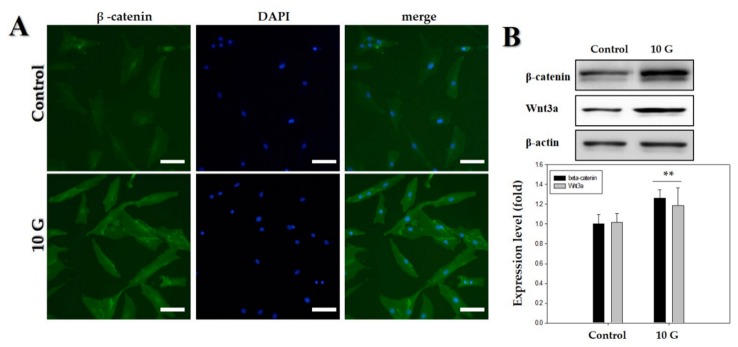
Upregulation of Wnt3α/β-catenin pathway by an extremely low-frequency electromagnetic field (ELF-EMF) with an intensity of 10 G. (**A**) Immunofluorescence staining of human hair follicle dermal papilla cells (hDPCs) after treatment with ELF-EMF; immunostaining with anti-β-catenin antibody (left panel). Corresponding DAPI nuclear staining is also shown (middle panel), and merged images are shown in the right panel. Original magnification was ×100; bar = 100 μm; (**B**) hDPCs were treated with ELF-EMF at an intensity of 10 G, and protein expression of Wnt3α/β-catenin after exposure to ELF-EMFs was analyzed by Western blotting and subsequent quantitative analysis. Expression levels of Wnt3α/β-catenin were analyzed in comparison with β-actin as the reference gene. ** *p* < 0.01, compared with control.

The hDPCs were treated with ELF-EMFs of 10 G intensity, and the expression of β-catenin and Wnt3α was measured via Western blot analysis and immunofluorescence staining. Treatment with an ELF-EMF of 10 G intensity increased the total level of Wnt3α/β-catenin protein expression (Figure 4).

### 2.4. ELF-EMF Effectively Promotes the Expression of Anagen-Related Molecules via Phosphorylation of GSK3β/ERK/AKT

**Figure 5:**
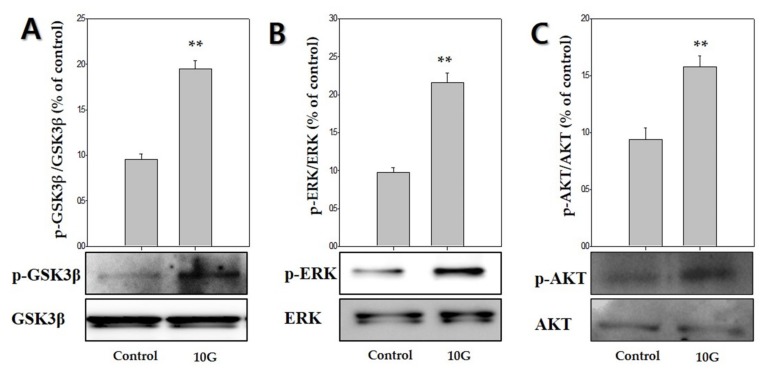
Extremely low-frequency electromagnetic fields (ELF-EMFs) stimulate anagen-related molecules by regulating the GSK3β/ERK/AKT signaling pathway. Protein levels were determined via Western blot analysis. (**A**) p-GSK3β expression; (**B**) p-ERK expression; (**C**) p-AKT expression. Each bar represents mean ± standard error of independent experiments performed in triplicate (*n* = 3). Significant differences were determined by the Student’s *t*-test; ** *p* < 0.01, compared with the control.

To determine whether GSK3β/ERK/AKT signaling was involved in ELF-EMF-induced proliferation of hDPCs and expression of anagen-related molecules. ELF-EMFs induced a significant increase in GSK3β/ERK/AKT phosphorylation (Figure 5). The p-GSK3β (Ser9) levels markedly increased by 1.9-fold after treatment with 10 G ELF-EMF (Figure 5A). ERK phosphorylation and AKT phosphorylation markedly increased by approximately 2.1-fold (Figure 5B) and 1.5-fold (Figure 5C), respectively. Thus, such data demonstrated that ELF-EMFs induced proliferation of hDPCs and expression of anagen-related molecules via GSK3β/ERK/AKT signaling-dependent pathway.

## 3. Discussion

The hDPCs regulate the development of human hair follicles, and the Wnt/β-catenin pathway is considered to be essential in maintaining hair-inducing activity of hDPCs [9]. To the best of our knowledge, the feasibility of using ELF-EMF on hDPCs has not yet been published. ELF-EMFs have been reported to have biological effects by impinging on cell viability and cell proliferation [29,30]. Moreover, EMFs have the advantage of being nontoxic and noninvasive.

This study aimed to determine the impact of ELF-EMF intensity on hDPCs and identify optimal conditions for ELF-EMF-induced expression of anagen-related molecules in hDPCs. We first examine the cytotoxic response of ELF-EMFs. ELF-EMF intensity-dependent cytotoxicity was evaluated by cell morphology and LDH assay. ELF-EMFs do not induce damage to the cell membrane of hDPCs, as verified via lactate dehydrogenase release assay (Figure 2A). Additionally, cell proliferation level was measured via MTT assay, and it was found that ELF-EMF intensities of 5, 10, 20, and 50 G significantly increased hDPC proliferation (Figure 2B). In particular, 10 G ELF-EMFs may positively impact the promotion of hDPC proliferation. The results of this study have revealed that the treatment of hDPCs with ELF-EMFs promoted cell proliferation and expression of anagen-related molecules without causing cell damage.

Versican and ALP are key characteristic molecular markers of DPs, which are specifically expressed in DPs during the anagen phase and absent during the telogen phase [8,31,32,33,34]. ALPs are expressed in DPCs throughout the hair growth cycle, ALP expression is often measured for determining the number and distribution of DPCs [26]; moreover, laminin represents the extracellular matrix present within DPs [31]. As shown in Figure 3A,B, anagen-related protein expression, versican, ALP, laminin, and collagen IV were strongly upregulated by exposure to ELF-EMFs at an intensity of 10 G. ELF-EMF-induced proliferation of DPCs might occur as a result of the activation of anagen-related protein expression.

Therefore, the study involving investigation of the signaling pathway was conducted at an EMF intensity of 10 G. We hypothesized that ELF-EMFs would modulate Wnt3α/β -catenin signaling in cultured hDPCs because the Wnt3α/β-catenin-mediated signaling pathway plays a pivotal role in the regulation of hair follicle morphogenesis, hair shaft differentiation, and follicular recycling [22,35,36]. Recent studies have demonstrated that DPCs possess β-catenin activity during their phase of active growth (i.e., anagen phase) [37] and that ablation of β-catenin in DPCs results in premature induction of the catagen phase [25]. It is also shown that the maintenance of β-catenin activity allows the cultured DPCs to retain the characteristics of their anagen phase [22,38]. These results show a correlation between β-catenin activity of DPCs and anagen duration and strongly suggest that the active growth-involving anagen phase can be prolonged by maintaining/activating β-catenin activity of DPCs. As shown in Figure 4A,B, the results revealed a significant increase in the activation of Wnt3a/β-catenin at an ELF-EMF intensity of 10 G. ELF-EMF-induced hDPC proliferation might occur as a result of the activation of Wnt3a/β-catenin.

To clarify the mechanisms by which ELF-EMF promotes the activity of Wnt3α/β-catenin in hDPCs, we examined changes in the expression of the GSK3β/ERK/AKT signaling pathway. GSK3β is inactivated by phosphorylation at Ser9. Thus, these results suggested that the ELF-EMF-induced increase in β-catenin protein levels occurring in hDPCs may be due to the reduced destruction caused by GSK3β inactivation. Moreover, Wnt3α/β-catenin signaling is involved in regulating the expression of ERK and PI3K/AKT, as well as the proliferation of hDPCs [27,28]. AKT promotes the phosphorylation of GSK-3β Ser9 and deactivates GSK-3β. The β-catenin is preserved due to deactivated GSK-3β, which causes cyclin D1 to manifest and provide positive feedback for the progress of the G1 phase. Cyclin D1 regulates cell proliferation by Wnt/β-catenin signaling [39,40,41]. Our results showed that treatment of hDPCs with ELF-EMFs at an intensity of 10 G significantly induced GSK/ERK/AKT phosphorylation, which also led to hDPCs’ proliferation and Wnt3α/β-catenin activation (Figure 5).

## 4. Materials and Methods

### 4.1. Cell Culture

The hair remaining after hair transplantation was collected after obtaining approval from the Institutional Review Board of the Dongguk University (DUIRB approval No. 20151127-011, 27 November 2015). Hair follicles were also obtained after the hair transplantation surgery, with the patients’ approval. The tissues were washed in Dulbecco’s phosphate balanced solution (DPBS) supplemented with penicillin, streptomycin, and amphotericin B. Hair follicles were delivered to the laboratory in Williams E medium, containing 200 unit/mL penicillin G, 0.2 mg/mL streptomycin, and 0.5 μg/mL amphotericin B.

Using a stereoscope at 40× magnification (KSZ, Korea), we sliced off the hair follicle bulb and used a 26 G syringe needle to isolate dermal papilla (DP) and dermal sheath (DS) by softly teasing the papilla. Subsequently, the basal stalk was cut off, and the dermal sheath cells (DSCs) were isolated. Finally, the papilla was transferred to a 35 mm culture dish that had been precoated with 400 µL of FBS, and then Dulbecco’s minimal essential medium (DMEM, 2 mL) was added, to reach a final concentration of 20% FBS. The media was fixed, while the cells grew from the attached tissue. Once the grown cells could be observed, FBS concentration was reduced to 10%. The media was changed twice a week, and 0.05% trypsin/0.02% EDTA solution was used to subculture confluent cells.

All cell media were replaced every 3 days. DPCs were subcultured when they reached a confluence of 80–90% in a 100Φ culture dish, with subculturing usually performed between 1 and 3 times. The cells were washed with phosphate-buffered saline (PBS), to remove FBS, dissociated using Accutase (Innovative Cell Technologies, San Diego, CA, USA), and then incubated at 37 °C, in a humid atmosphere of 5% CO_2_, for 5–7 min. Next, the Accutase was collected and removed from the cells by centrifugation at 800 rpm for 5 min. The supernatant was discarded, and the cell suspension was inoculated into a fresh cell culture medium (DMEM containing 10% FBS, 100 unit/mL of penicillin G, 0.1 mg/mL of streptomycin, and 0.25 μg/mL of amphotericin B) and seeded in a 100Φ culture dish.

### 4.2. EMF Exposure

**Figure 6:**
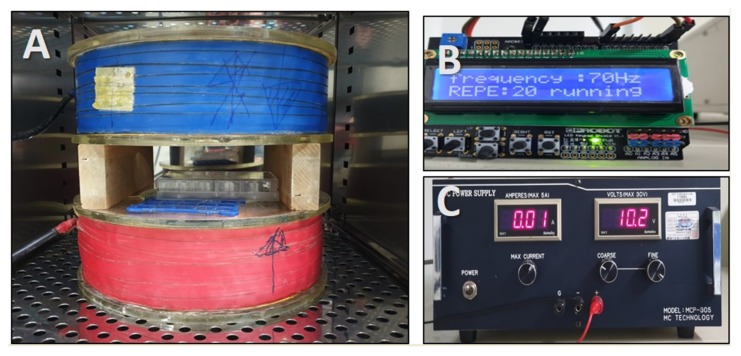
Photograph of the extremely low-frequency electromagnetic field (ELF-EMFEMF) device placed in an incubator. ELF-EMF was generated by using a pair of Helmholtz coils. (**A**) Helmholtz coil, (**B**) function generator, and (**C**) power supply.

We used a Helmholtz coil, which can generate magnetic fields; the apparatus is depicted in Figure 6. The stimulus frequency was 70 Hz, and the stimulus wave was in pulse form. The electromagnetic field device was placed in an incubator, at 37 °C and 5% humidified atmosphere, and hDPCs were stimulated by ELF-EMFs, at frequencies of 5, 10, 20, 50, and 100 G, for 15 min, each day, for 4 days. Controls were cultured at a separate location, to avoid exposure to ELF EMFs.

### 4.3. Cell Proliferation Assay

Cell proliferation was determined by using an MTT assay. Briefly, hDPCs were seeded at a density of 2 × 10^4^ cells/well into 24-well plates. After 24 h of incubation, hDPCs irradiated with EMF. After 4 days, the cells were treated with an MTT reagent for 4 h. The samples were assessed by measuring absorbance at 540 nm with an ELISA plate reader. Cytotoxicity was evaluated by the LDH assay; the medium was collected after cell culture, and an LDH-LQ kit was used (Asan Pharmaceutical, Seoul, Korea). The medium (100 μL) was placed in a 96-well plate, with 50 μL of working solution, and incubated at room temperature for 30 min. Then, the reaction was terminated with 50 μL of stop solution (1 N HCl), and the absorbance was measured at 570 nm.

### 4.4. Western Blot Analysis

Cells were washed with PBS, extracted with sample buffer (pH 6.8) consisting of 5% 2-mercaptoethanol, 10% glycerol, and 0.1 mg/mL bromophenol blue dissolved in Tris-HCl, and boiled at 100 °C for 5 min. To load the same amount of protein, BCA assay was performed, and the same amount of protein (20–40 μg) was separated via electrophoresis, on 8–10% SDS-PAGE, and transferred onto a nitrocellulose membrane. Subsequently, these separated proteins were incubated with primary antibodies recognizing β-actin (Sigma-Aldrich, St Louis, MO, USA), type IV collagen, laminin, β-catenin, Wnt3α, versican, alkaline phosphatase (ALP), p-GSK3β, GSK3β, p-ERK, ERK, p-AKT, and AKT (Abcam, Cambridge, MA, USA). Protein detection was performed with Lumi Femto (Daeil Laboratory Service, Seoul, Korea) and the Molecular Imager ChemiDoc XRS+ station. ImageJ software (National Institutes of Health, Bethesda, MD, USA) was used to analyze and quantify the Western blot image.

Band images were obtained by using the ECL system (Thermo Fisher Scientific, Waltham, MA, USA) and Molecular Imager ChemiDoc XRS+ (Bio-Rad, Hercules, CA, USA). ImageJ software was used for the quantitative analysis of Western blot band images.

### 4.5. Immunofluorescent Staining Analysis

The cells were washed with PBS before being fixed in 4% paraformaldehyde prepared in PBS. Cells were immunostained according to standard protocols, using the following primary antibodies: β-catenin (Abcam) and appropriate fluorescent secondary antibodies (cell signaling). Representative images were captured by using a Nikon Eclipse Ti microscope.

### 4.6. Statistical Analysis

All data are presented as the mean ± standard deviation (SD) of three independent experiments. The n-values indicate the number of independent experiments performed or the number of individual experiments. For each independent experiment, at least three technical replicates were used and a minimum number of three independent experiments were performed to ensure adequate statistical power. In all of the analyses, group differences were considered to be statistically significant at *p* < 0.05 (* *p* < 0.05, ** *p* < 0.01). An ANOVA test was used for multicomponent comparisons, and the Student’s t-test for two-component comparisons after normal distribution was confirmed.

## 5. Conclusions

Our results suggest that ELF-EMFs have the potential to stimulate anagen-related molecules in hDPCs, as well as hDPC proliferation via the activation of Wnt3α/β-catenin signaling and GSK-3β/ERK/Akt pathway. To the best of our knowledge, this is the first report to demonstrate the effect of various ELF-EMF intensities on anagen-related molecule expression in hDPCs and the underlying mechanisms. Our study also reports the optimal intensity of ELF-EMFs on hDPC proliferation and anagen-related molecule expression. However, considering that the human hair follicle is a complex organ composed of epithelial and mesenchymal tissues and that the interaction of these tissues is essential for hair follicle growth, more studies on human hair follicle organ culture or in vivo studies are warranted. Taken together, these results strongly suggest that an ELF-EMF intensity of 10 G may prolong the anagen phase in hDPCs by increasing type IV collagen, ALP, and versican levels via activation of Wnt3α/β-catenin signaling and phosphorylation of GSK3β/ERK/AKT. Therefore, it is considered that ELF-EMF may be particularly useful for hair loss prevention and hair growth enhancement.

## References

[1] Madaan A., Verma R., Singh A.T., Jaggi M. (2018). Review of Hair Follicle Dermal Papilla cells as in vitro screening model for hair growth. Int. J. Cosmetic Sci..

[2] Wen T.C., Li Y.S., Rajamani K., Harn H.J., Lin S.Z., Chiou T.W. (2018). Effect of Cinnamomum osmophloeum Kanehira Leaf Aqueous Extract on Dermal Papilla Cell Proliferation and Hair Growth. Cell Transplant..

[3] Boisvert W.A., Yu M., Choi Y., Jeong G.H., Zhang Y.L., Cho S., Choi C., Lee S., Lee B.H. (2017). Hair growth-promoting effect of Geranium sibiricum extract in human dermal papilla cells and C57BL/6 mice. BMC Complement. Altern. Med..

[4] Nam G.H., Jo K.J., Park Y.S., Kawk H.W., Yoo J.G., Jang J.D., Kang S.M., Kim S.Y., Kim Y.M. (2019). Bacillus/Trapa japonica Fruit Extract Ferment Filtrate enhances human hair follicle dermal papilla cell proliferation via the Akt/ERK/GSK-3beta signaling pathway. BMC Complement. Altern. Med..

[5] Kim Y.M., Kwon S.J., Jang H.J., Seo Y.K. (2017). Rice bran mineral extract increases the expression of anagen-related molecules in human dermal papilla through wnt/catenin pathway. Food Nutr. Res..

[6] (2016). Effect of PDGF-C on biological characters of human dermal papilla cells in vitro. Zhonghua zheng xing wai ke za zhi = Zhonghua zhengxing waike zazhi = Chin. J. Plastic Surg..

[7] Kiso M., Hamazaki T.S., Itoh M., Kikuchi S., Nakagawa H., Okochi H. (2015). Synergistic effect of PDGF and FGF2 for cell proliferation and hair inductive activity in murine vibrissal dermal papilla in vitro. J. Dermatol. Sci..

[8] Lee A., Bae S., Lee S.H., Kweon O.K., Kim W.H. (2017). Hair growth promoting effect of dermal papilla like tissues from canine adipose-derived mesenchymal stem cells through vascular endothelial growth factor. J. Vet. Med. Sci..

[9] Jampa-Ngern S., Viravaidya-Pasuwat K., Suvanasuthi S., Khantachawana A. (2017). Effect of laser diode light irradiation on growth capability of human hair follicle dermal papilla cells. Proceedings of the 2017 39th Annual International Conference of the IEEE Engineering in Medicine and Biology Society (EMBC).

[10] Lee S.W., Juhasz M., Mobasher P., Ekelem C., Mesinkovska N.A. (2018). A systematic review of topical finasteride in the treatment of androgenetic alopecia in men and women. J. Drugs Dermatol..

[11] Arca E., Açıkgöz G., Taştan H.B., Köse O., Kurumlu Z.J.D. (2004). An open, randomized, comparative study of oral finasteride and 5% topical minoxidil in male androgenetic alopecia. Dermatology.

[12] Sica D.A. (2004). Minoxidil: An underused vasodilator for resistant or severe hypertension. J. Clin. Hypertens..

[13] Seidman S.J., Guag J.W. (2013). Adhoc electromagnetic compatibility testing of non-implantable medical devices and radio frequency identification. Biomed. Eng. Online.

[14] Tasset I., Pérez-Herrera A., Medina F.J., Arias-Carrión Ó., Drucker-Colín R., Túnez I. (2013). Extremely low-frequency electromagnetic fields activate the antioxidant pathway Nrf2 in a Huntington’s disease-like rat model. Brain Stimul..

[15] Gaetani R., Ledda M., Barile L., Chimenti I., De Carlo F., Forte E., Ionta V., Giuliani L., D’Emilia E., Frati G. (2009). Differentiation of human adult cardiac stem cells exposed to extremely low-frequency electromagnetic fields. Cardiovasc. Res..

[16] Mayer-Wagner S., Passberger A., Sievers B., Aigner J., Summer B., Schiergens T.S., Jansson V., Müller P.E. (2011). Effects of low frequency electromagnetic fields on the chondrogenic differentiation of human mesenchymal stem cells. Bioelectromagnetics.

[17] Kim H.-J., Jung J., Park J.-H., Kim J.-H., Ko K.-N., Kim C.-W. (2013). Medicine, Extremely low-frequency electromagnetic fields induce neural differentiation in bone marrow derived mesenchymal stem cells. Exp. Biol. Med..

[18] Piacentini R., Ripoli C., Mezzogori D., Azzena G.B., Grassi C. (2008). Extremely low-frequency electromagnetic fields promote in vitro neurogenesis via upregulation of Cav1-channel activity. J. Cell. Physiol..

[19] Yan J., Dong L., Zhang B., Qi N. (2010). Effects of extremely low-frequency magnetic field on growth and differentiation of human mesenchymal stem cells. Electromagn. Biol. Med..

[20] Bureau J., Ginouves P., Guilbaud J., Roux M.J.A. (2003). Essential oils and low-intensity electromagnetic pulses in the treatment of androgen-dependent alopecia. Adv. Ther..

[21] Sun Y.N., Cui L., Li W., Yan X.T., Yang S.Y., Kang J.I., Kang H.K., Kim Y.H. (2013). Promotion effect of constituents from the root of Polygonum multiflorum on hair growth. Bioorg. Med. Chem. Let..

[22] Kishimoto J., Burgeson R.E., Morgan B.A. (2000). Wnt signaling maintains the hair-inducing activity of the dermal papilla. Gene. Dev..

[23] Chu E.Y., Hens J., Andl T., Kairo A., Yamaguchi T.P., Brisken C., Glick A., Wysolmerski J.J., Millar S.E. (2004). Canonical WNT signaling promotes mammary placode development and is essential for initiation of mammary gland morphogenesis. Development.

[24] Jo S.J., Choi S.-J., Yoon S.-Y., Lee J.Y., Park W.-S., Park P.-J., Kim K.H., Eun H.C., Kwon O. (2013). Valproic acid promotes human hair growth in in vitro culture model. J. Dermatol. Sci..

[25] Enshell-Seijffers D., Lindon C., Kashiwagi M., Morgan B.A. (2010). β-catenin activity in the dermal papilla regulates morphogenesis and regeneration of hair. Dev. Cell.

[26] Park P.J., Moon B.S., Lee S.H., Kim S.N., Kim A.R., Kim H.J., Park W.S., Choi K.Y., Cho E.G., Lee T.R. (2012). Hair growth-promoting effect of Aconiti Ciliare Tuber extract mediated by the activation of Wnt/beta-catenin signaling. Life Sci..

[27] Hwang K.-A., Hwang Y.-L., Lee M.-H., Kim N.-R., Roh S.-S., Lee Y., Kim C.D., Lee J.-H., Choi K.-C. (2012). Adenosine stimulates growth of dermal papilla and lengthens the anagen phase by increasing the cysteine level via fibroblast growth factors 2 and 7 in an organ culture of mouse vibrissae hair follicles. Int. J. Mol. Med..

[28] Yoon S.-Y., Yoon J.-S., Jo S.J., Shin C.Y., Shin J.-Y., Kim J.-I., Kwon O., Kim K.H. (2014). A role of placental growth factor in hair growth. J. Dermatol. Sci..

[29] Hernández-Pavón J.C., Sosa M., Córdova T., Barbosa-Sabanero G., Solorio-Meza S., Sabanero-López M. (2009). Study of Electromagnetic Fields on Cellular Systems. Acta Univ..

[30] Lee H.C., Hong M.N., Jung S.H., Kim B.C., Suh Y.J., Ko Y.G., Lee Y.S., Lee B.Y., Cho Y.G., Myung S.H. (2015). Effect of extremely low frequency magnetic fields on cell proliferation and gene expression. Bioelectromagnetics.

[31] Müntener T., Doherr M.G., Guscetti F., Suter M.M., Welle M.M. (2011). The canine hair cycle–a guide for the assessment of morphological and immunohistochemical criteria. Vet. Dermatol..

[32] Yang C.-C., Cotsarelis G. (2010). Review of hair follicle dermal cells. J. Dermatol. Sci..

[33] Du Cros D.L., LeBaron R.G., Couchman J.R. (1995). Association of versican with dermal matrices and its potential role in hair follicle development and cycling. J. Investig. Dermatol..

[34] Kishimoto J., Ehama R., Wu L., Jiang S., Jiang N., Burgeson R.E. (1999). Selective activation of the versican promoter by epithelial–mesenchymal interactions during hair follicle development. Proc. Natl. Acad. Sci. USA.

[35] Kitagawa T., Matsuda K., Inui S., Takenaka H., Katoh N., Itami S., Kishimoto S., Kawata M. (2009). Keratinocyte growth inhibition through the modification of Wnt signaling by androgen in balding dermal papilla cells. J. Clin. Endocrinol. Metab..

[36] Andl T., Reddy S.T., Gaddapara T., Millar S.E. (2002). WNT signals are required for the initiation of hair follicle development. Dev. Cell.

[37] Maretto S., Cordenonsi M., Dupont S., Braghetta P., Broccoli V., Hassan A.B., Volpin D., Bressan G.M., Piccolo S. (2003). Mapping Wnt/β-catenin signaling during mouse development and in colorectal tumors. Proc. Natl. Acad. Sci. USA.

[38] Shimizu H., Morgan B.A. (2004). Wnt signaling through the β-catenin pathway is sufficient to maintain, but not restore, anagen-phase characteristics of dermal papilla cells. J. Investig. Dermatol..

[39] Joo H.J., Jeong K.H., Kim J.E., Kang H. (2017). Various wavelengths of light-emitting diode light regulate the proliferation of human dermal papilla cells and hair follicles via Wnt/β-catenin and the extracellular signal-regulated kinase pathways. Ann. Dermatol..

[40] Aubry J.-M., Schwald M., Ballmann E., Karege F.J.P. (2009). Early effects of mood stabilizers on the Akt/GSK-3β signaling pathway and on cell survival and proliferation. Psychopharmacology.

[41] Dent P., Yacoub A., Fisher P.B., Hagan M.P., Grant S.J.O. (2003). MAPK pathways in radiation responses. Oncogene.

